# Modeling uncertainty: the impact of noise in T cell differentiation

**DOI:** 10.3389/fsysb.2024.1412931

**Published:** 2024-08-06

**Authors:** David Martínez-Méndez, Carlos Villarreal, Leonor Huerta

**Affiliations:** ^1^ Instituto de Investigaciones Biomédicas, Departamento de Inmunología, Universidad Nacional Autónoma de México, Mexico City, Mexico; ^2^ Instituto de Física, Universidad Nacional Autónoma de México, Mexico City, Mexico

**Keywords:** CD4 T lymphocytes, noise, cytokines, complex network, differentiation, stochastic process, hypoxia, glutamine

## Abstract

**Background:**

The regulatory mechanisms guiding CD4 T cell differentiation are complex and are further influenced by intrinsic cell variability along with that of microenvironmental cues, such as cytokine and nutrient availability.

**Objective:**

This study aims to expand our understanding of CD4 T cell differentiation by examining the influence of intrinsic noise on cell fate.

**Methodology:**

A model based on a complex regulatory network of early signaling events involved in CD4 T cell activation and differentiation was described in terms of a set of stochastic differential equation to assess the effect of noise intensity on differentiation efficiency to the Th1, Th2, Th17, Treg, and ${\text{T}}_{FH}$ effector phenotypes under defined cytokine and nutrient conditions.

**Results:**

The increase of noise intensity decreases differentiation efficiencies. In a microenvironment of Th1-inducing cytokines and optimal nutrient conditions, noise levels of 3 % , 5 % and 10 % render Th1 differentiation efficiencies of 0.87, 0.76 and 0.62, respectively, underscoring the sensitivity of the network to random variations. Further increments of noise reveal that the network is relatively stable until noise levels of 20 % , where the resulting cell phenotypes becomes heterogeneous. Notably, Treg differentiation showed the highest robustness to noise perturbations. A combined Th1-Th2 cytokine environment with optimal nutrient levels induces a dominant Th1 phenotype; however, removal of glutamine shifts the balance towards the Th2 phenotype at all noise levels, with an efficiency similar to that obtained under Th2-only cytokine conditions. Similarly, combinations of Th1/Treg and Treg/Th17-inducing cytokines along with the removal of either tryptophan or oxygen shift the dominant Th1 and Treg phenotypes towards Treg and Th17 respectively. Model results are consistent with differentiation efficiency patterns obtained under well-controlled experimental settings reported in the literature.

**Conclusion:**

The stochastic CD4 T cell mathematical model presented here demonstrates a noise-dependent modulation of T cell differentiation induced by cytokines and nutrient availability. Modeling results can be explained by the network topology, which assures that the system will arrive at stable states of cell functionality despite variable levels of biological intrinsic noise. Moreover, the model provides insights into the robustness of the T cell differentiation process.

## 1 Introduction

### 1.1 Stochastic mathematical models in biology

Biological systems are epitomes of complexity and dynamism, often exhibiting behaviours that are both unpredictable and highly regulated. Emergent biological properties arise from individual interactions among the system components (nodes) leading to system self-organization and function (Anderson, 1972; Glass and Kauffman, 1973; Haken, 1978; Kauffman, 1992). Modeling the intricate interplay of genetic, environmental, and internal fluctuating elements within these systems can be achieved through stochastic mathematical models capturing the inherent randomness and nuances of biological processes. Unlike deterministic models, stochastic models incorporate randomness directly into equations, and iterative analysis produces a range of possible outcomes and their associated probabilities (Elowitz et al., 2002; Swain et al., 2002). The use of stochastic models in biology allows the analysis of the consequences of intrinsic noise on robustness of emergent properties, and thus, system stability despite internal and external fluctuations (Haken, 1978). This property has been extensively explored in the context of biological systems, revealing how they might achieve resilience (Barkai and Leibler, 1997; Kitano, 2007). Moreover, stochastic models have been instrumental in exploring system stability and bifurcation points, where small changes in parameters can lead to drastic shifts in system behavior. This concept is especially relevant in immunology, since the immune system must adapt to a vast array of pathogens and microenvironmental conditions while preventing excessive or insufficient responses. These considerations underscore the importance of mathematical modeling in predicting disease progression by understanding immune system dynamics, and identifying potential therapeutic interventions (Tyson and Novák, 2013).

The adaptive immune system’s response to pathogens relies on the differentiation of CD4 T cells into various effector phenotypes, such as Th1, Th2, Th17, Treg, and ${\text{T}}_{FH}$ , which are crucial for mounting effective immune responses and maintaining immunological balance. A model of the early intracellular events arising upon T-cell receptor (TCR) and CD28 signaling leading to function has been previously built by incorporating overarching principles generated by experimental research into a network of interactions between nodes. The network architecture was originally described by means of dichotomous Boolean logical rules (Martínez-Méndez et al., 2020) and then it was straightforwardly translated into continuous fuzzy-logic rules, allowing the description of the temporal evolution of the network (Martínez-Méndez et al., 2021). The model yielded outcomes of activation, differentiation, regulation and metabolic changes which qualitatively reproduce general patterns revealed by a number of experimental investigations. Of particular interest is the observation that a cell microenvironment constituted by an all-type mixture of exogenous cytokines (IFN-g, IL-12, IL-4, IL-18, IL-33, TGF- β , IL-10, IL-21 and IL-6) along with particular levels of nutrients (glutamine and tryptophan) and oxygen, may induce a hierarchy of phenotype’s expression. When an all-type cytokine mixture, nutrients, and oxygen are present at optimal concentration, the network dynamics leads the system to a predominant Th1 polarization. By systematically decreasing the level of the corresponding lineage-defining cytokines or nutrients, a hierarchical pattern of phenotype expressions was revealed that may be represented as a transition sequence: Th1 → Th2 → Th17 → Treg (Martínez-Méndez et al., 2022). In this work, we study the effects of fluctuations (noise) of endogenous and exogenous interactions on T CD4 cell differentiation in the same set of microenviromental conditions mentioned before. Fluctuations may arise from random variations of the exogenous cell microenvironment or inner signalling pathways. For example, T cell function can be controlled by the metabolic programmes of the cell that respond dynamically to fluctuations in the nutrients, oxygen levels and energy sources during migration between distinct microenvironments, such as between lymphoid organs and tissues or tumour sites (Pearce et al., 2013).

**FIGURE 1:**
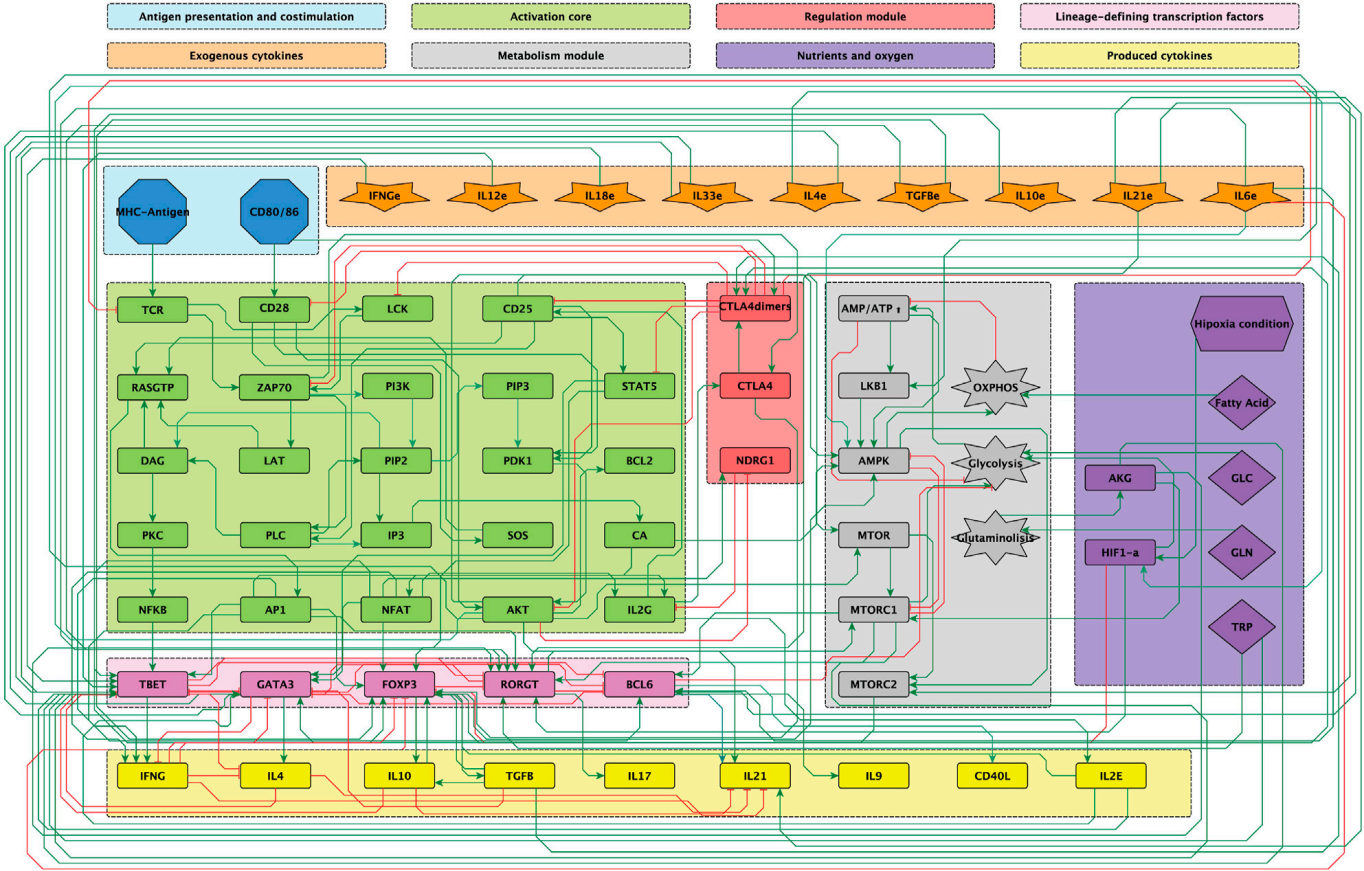
Modular 68-node network of early events in CD $4^{+}$ T cell activation, differentiation, metabolic activity and response to nutrients. Modules are described in the upper panel and their respective elements are indicated with the corresponding color in the network. Green lines represent activating connections. Red lines represent inhibitory connections. Taken from Martínez-Méndez et al. (2022).

**FIGURE 2:**
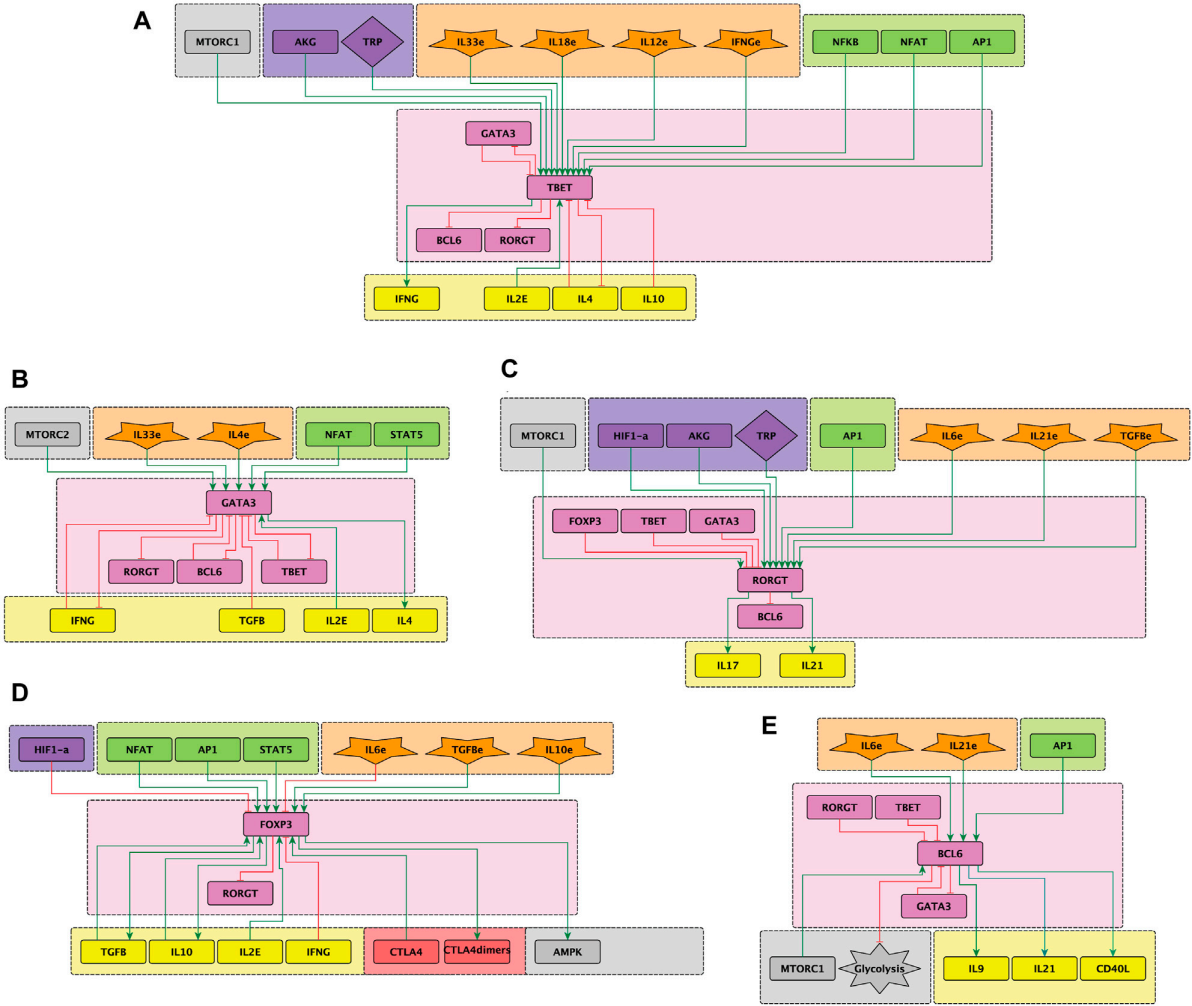
Sub-networks representing the effect of exogenous cytokines (orange) and signaling from transcription factors and metabolism elements (green and grey) on the activity of the lineage-defining transcription factors (LTF’s) (pink) leading to differentiaton of CD4 T cells towards effector phenotypes. **(A)** T-bet, **(B)** GATA3, **(C)** ROR γ t, **(D)** Foxp3, **(E)** Bcl-6. Exogenous cytokines induce the activity of LTF’s, which tightly regulate each other (T-bet, GATA3, ROR γ t, Foxp3 and BCL-6) (pink), and guide cell differentiation towards effector cell subtypes (Th1, Th2, TH17, Treg and ${\text{T}}_{FH}$ , respectively). Cytokines produced by differentiated cells are indicated (yellow). Nutrients and oxygen levels (violet) can directly modulate the activity of LTF’s or alter the metabolic equilibrium of lymphocytes, strongly influencing cell differentiation.

We generalized the previously employed methodology by introducing a set of stochastic differential equations (Langevin equations), allowing the analysis of different levels of noise on cell fate (Villarreal et al., 2012), so that we explore how intrinsic noise influences the robustness and adaptability of the immune response. The regulatory network considered before (Martínez-Méndez et al., 2022) has been expanded here by incorporating the dendritic cell phase of ${\text{T}}_{FH}$ differentiation, as shown in Figure 2. As a whole, the network embraces the signaling from the TCR and co-stimulatory molecules, cell metabolism regulators and the tightly regulated interactions among lineage-defining transcription factors (LTF’s), as displayed in Figures 1, 2.

## 2 Material and methods

### 2.1 CD4 T cell signaling network inference and modular organization

Figure 1 shows the signalling network of interactions involved in the activation of CD4 lymphocytes upon binding of TCR-specific antigen and co-stimulatory molecules. It incorporates interactions associated to microenvironmental nutrients (glutamine and tryptophan), hypoxia and anti-inflammatory drugs. The network includes the inducers of CD4 T cell activation leading to differentiation of naïve CD4 T cells into several types of effector cells (inputs): antigenic stimulation, co-stimulation and the activity of cytokines in the microenvironment. Specific exogenous cytokines promote the activity of intracellular lineage-defining transcription factors (LTF’s) directing cell differentiation by, in turn, inducing the production of lymphocyte-derived cytokines. Thus, Th1 effector cells are induced by IL-12 and IFN- γ , express the T-bet transcription factor, and produce IFN- γ . Th2 cells require IL-4 and are stabilized by IL-2, express GATA3, and produce IL-4, IL-5, and IL-13. Th17 cells require TGF- β and IL-6, IL-21, or IL-23, express ROR γ t, and produce IL-21, IL-17A, and IL-17F. Treg cells require TGF- β and IL-2, express Foxp3, and produce TGF- β and IL-10 in some cases. Very often, however, immunological challenges bring about a variety of cytokines that do not necessarily match definite patterns inducing particular effector phenotypes. Rather, the presence of infectious agents, particularly during their persistence in the host, induces the production of mixtures of cytokines that, in principle, may act synergistically or antagonistically during differentiation to effector phenotypes (reviewed in (Dupage and Bluestone, 2016)). The network involves 68 nodes organized in eight modules. Three modules correspond to inputs or entries of the system: 1) antigen presentation and co-stimulation, 2) phenotype-inducing microenvironmental cytokines, and 3) nutrient and oxygen availability; four modules represent intracellular interactions leading to activation and production of cytokines, including 4) an activation core containing signals induced downstream the TCR and CD28, as well as pathways leading to the synthesis of IL-2 and the expression of CD25, the alpha chain providing high affinity to the IL-2 receptor complex; 5) a metabolic regulation module, with the nutrient sensor AMPK acting as the central regulator of glycolysis and OXPHOS along with mTOR elements, and 6) a regulatory module including the activity of CTLA-4 and the anergy factor NDRG1; 7) a module corresponding to the expression of the lineage-defining transcription factors T-bet, GATA3, Foxp3, ROR γ t and Bcl-6 (Figure 2), and 8) a module including the output cytokines produced by differentiated cells. The modular organization for the graphical display of the network allows for the rapid identification of the node’s main role. Detailed diagrams of sub-networks and bibliography used for network construction can be found in (Martínez-Méndez et al., 2020; Martínez-Méndez et al., 2021; Martínez-Méndez et al., 2022).

### 2.2 Stochastic regulatory networks

**FIGURE 3:**
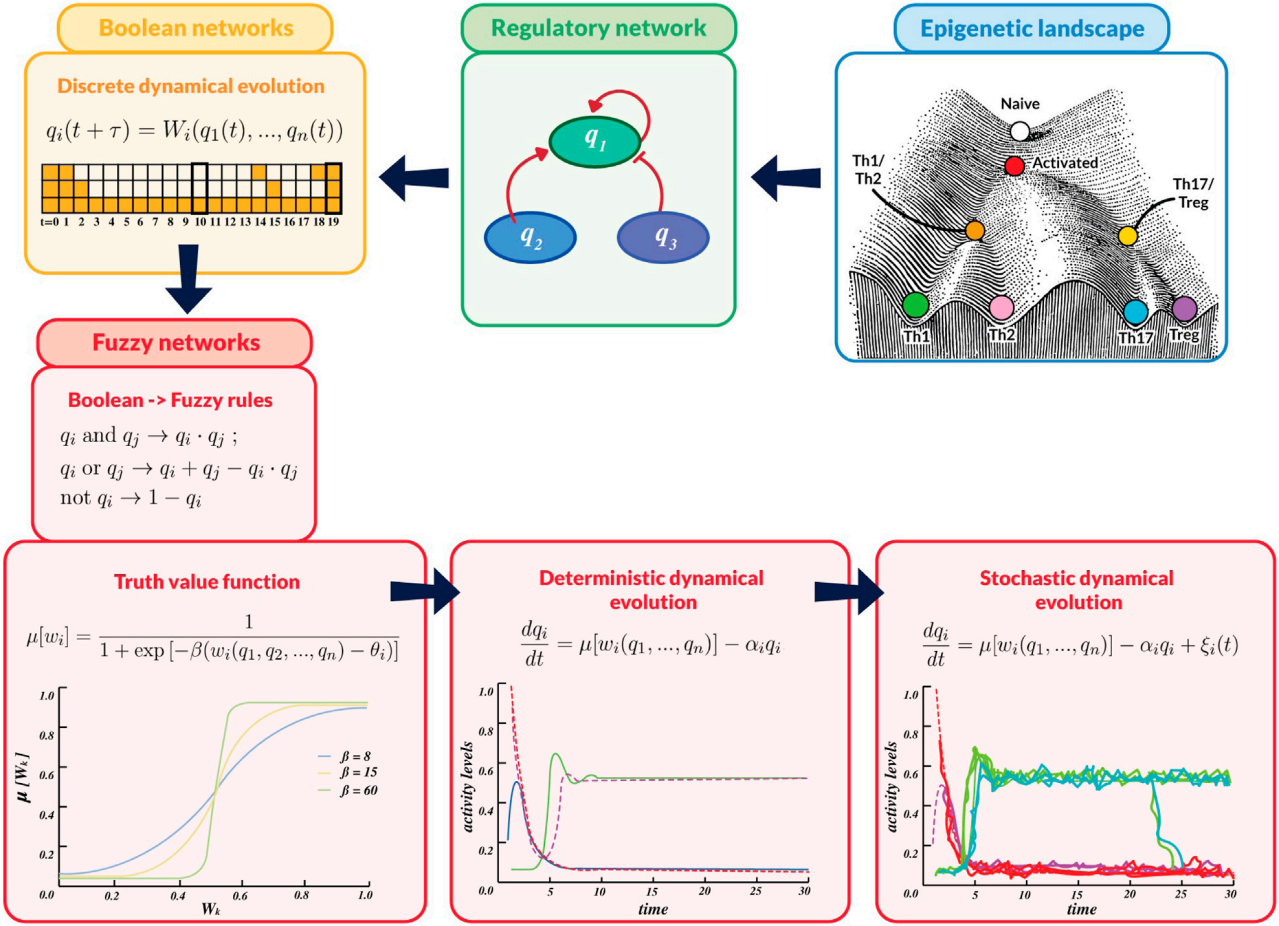
Flux diagram summarizing the mathematical methodology used in this work. An overall assumption is that i) cell processes in developmental biology comply with logical rules that can be described by a network of node interactions, underlying the epigenetic landscape. ii) These rules are expressed in terms of Boolean propositions representing nodes interactions, giving rise to iii) discrete-time dynamics whose steady states define cell phenotypes. The variables, logic rules and dynamic description are then translated into a continuous scheme by iv) introducing fuzzy-logic rules, $w_{i}$ . v) Truth functions, $\mu \left[w_{i}\right]$ , are defined by means of sigmoid expressions incorporating the fuzzy rules. $\mu \left[w_{i}\right]$ shows a continuous variation with values ranging between 0 (unexpressed) and 1 (fully expressed). vi) The system dynamics is described through a set of ordinary differential equations (ODE), with inputs defined by the fuzzy rules. vii) The influence of noise on the network dynamics is studied by introducing stochastic perturbations into the set of ODE.

The formal methodology discussed below has been summarized in the flux diagram depicted in Figure 3. The use of complex networks in Systems Biology allows the construction of a conceptual framework of endogenous and exogenous interactions defining signalling pathways involved in cell function (Albert and Thakar, 2014). A regulatory network is constructed by connected nodes and their inner relations where every node represents a gene, a transcription factor, a cytokine, etc. In its most basic approximation, the expression value of node i is characterized by a discrete variable, $q_{i}$ , which may acquire either the value, 0 or 1, while the node interactions are described by Boolean logical propositions. A more accurate investigation may be performed by considering a continuous logical analysis where the expression values of the network variables $q_{i}$ display a continuous variation with truth values ranging within a continuous range (between 0 and 1) limited only by functionality constraints. This formalism, termed as fuzzy logic, incorporates multi-valued propositions that allow to represent, manipulate, and interpret imprecise or vague information, and has the capability of implementing a well-defined inference scheme (Zadeh, 1965). In previous works, we have employed this kind of scheme to analyze the activation and differentiation processes of CD4^+^ T cells and their modulation by micro-environmental conditions (Martínez-Méndez et al., 2021; Martínez-Méndez et al., 2022). Similarly, it has been used to study signalling pathways of pancreatic beta cells and the development of type-2 diabetes (Barrera et al., 2020).

In the fuzzy logic scheme, the network interactions are described by continuous logical propositions $w_{i}\left(q_{1},q_{2},\ldots ,q_{n}\right)$ in which binary Boolean operators are replaced by algebraic relations (Enciso et al., 2019):

$$q_{i}\;and\;q_{j}\to q_{i}\cdot q_{j}\;;\;\;\;\;\;q_{i}\;or\;q_{j}\to q_{i}+q_{j}-q_{i}\cdot q_{j}\;;\;\;\;\;\;not\;q_{i}\to 1-q_{i}.$$ (1)

For example, the Boolean relation,

$$\left(q_{i}\;or\;q_{j}\right)\;and\;\left(not\;q_{k}\right)\;\to \;\left(q_{i}+q_{j}-q_{i}\cdot q_{j}\right)\cdot \left(1-q_{k}\right).$$

The truth value of a fuzzy proposition $w_{i}$ is expressed as a categorical function, $\mu \left[w_{i}\right]$ , with a sigmoid structure. A possible choice is:

$$\mu \left[w_{i}\right]=\frac{1}{1+\exp\left[-\beta \left(w_{i}\left(q_{1},q_{2},\ldots ,q_{n}\right)-\theta_{i}\right)\right]},$$ (2)

where $\mu \left[w_{i}\right]$ may variate within the range $0\leq \mu \left[w_{i}\right]\leq 1$ . Its explicit value is determined by the difference $w_{i}-\theta_{i}$ where $\theta_{i}$ is an expression threshold, usually considered as $\theta_{i}=1/2$ , and the parameter β represents a saturation rate which is assumed as β=10 in this study. The dynamic evolution of the network expression values, $q_{i}$ , is now determined by a system of ordinary differential equations (ODEs) (Mendoza and Xenarios, 2006; Villarreal et al., 2012; Enciso et al., 2019):

$$\frac{dq_{i}}{dt}=\mu \left[w_{i}\left(q_{1},\ldots ,q_{n}\right)\right]-\alpha_{i}q_{i},$$ (3)

where $\alpha_{i}$ is the decay rate of node i , assumed in this study as a default value $\alpha_{i}=1$ . As is well-known, for any set of initial conditions, $\left\{q_{1}\left(0\right),\ldots ,q_{n}\left(0\right)\right\}$ , the ODE system (3) gives rise to dynamical trajectories $\left\{q_{1}\left(t\right),\ldots ,q_{n}\left(t\right)\right\}$ which are uniquely determined by this set. In the long-time limit the trajectories converge into a collection of r stable states, or attractors, ${\left\{q_{1}^{st},\ldots ,q_{n}^{st}\right\}}_{k}$ , with k=1,…,r . In the present study, the different attractor sets represent r alternative cell fates.

The fuzzy logic methodology allows to construct a conceptual framework concerning the mechanistic underlying the activation and differentiation of T cells. However, this approach considers that the intensity of the network interactions remain invariable under the time evolution of the system, thus representing a deterministic description. However, no biological process works with total certainty since it is always subject to the action of a number of unknown events. Thus, here we investigate the influence of fluctuations in the T cell differentiation process.

The effect of random perturbations on the cell functionality can be visualized by recurring to the metaphor of the epigenetic landscape (EL) proposed by C. Waddington to describe the way in which gene regulation modulates cell development (Waddington, 1957). In this view, cell fate is determined by the successive transit of states defined by EL basins until it reaches a steady state called attractor. This phenomenon can be compared with the pathway of a ball on a landscape where its initial position represents an initial stem cell state, and it moves through transient intermediate basins, until it reaches an equilibrium basin determining the final cell fate. Now, a stochastic EL can be introduced by considering that under the action of noise, the ball suffers random collisions along its epigenetic trajectory; depending on the noise intensity, this process could modify the final cell fate. A formal realization of the stochastic EL can be constructed by introducing a ‘white noise’ characterized by time-dependent random variables, $\xi_{i}\left(t\right)$ , with a Gaussian probability distribution, with a null average $\left\langle \xi_{i}\left(t\right)\right\rangle =0$ , and a very short time-correlation $\left\langle \xi_{i}\left(t\right)\;\xi_{i}\left(t^{\prime }\right)\right\rangle =2D\;\Phi \left(t-t^{\prime }\right)$ . Here, D is a diffusion coefficient representing a measure of the noise intensity, while Φ is a highly-peaked distribution at time $t=t^{\prime }$ , so that the white noise at a time t does not keep memory of its value at previous times $t^{\prime }$ (McQuarrie, 1976). In this characterization, the average of any random variable is evaluated by introducing the notion of a statistical ensemble. This concept contemplates a large set of identically prepared systems which however display an uncontrollable dispersion of their possible initial conditions. This construction may be either of theoretical or experimental character, this latter reflecting the fact that repetitions of carefully prepared experiments yield slightly different results in every measurement (Brody, 1992).

The effect of noise on the system dynamics can be studied by means of a set of Langevin stochastic differential equations (Chandrasekhar, 1973):

$$\frac{dq_{i}}{dt}=\mu \left[w_{i}\left(q_{1},\ldots ,q_{n}\right)\right]-\alpha_{i}q_{i}+\xi_{i}\left(t\right).$$ (4)

The equilibrium state my be derived by considering the steady-state condition, $\left\langle dq_{i}/dt\right\rangle =0$ , leading to

$$\langle q_{i}^{st}\rangle =\frac{1}{\alpha_{i}}\langle \mu \left[w_{i}\left(q_{1}^{st},\ldots ,q_{n}^{st}\right)\right]\rangle .$$ (5)

We observe that, since $\alpha_{i}=1/\tau_{i}$ , where $\tau_{i}$ is the characteristic decay time, this relation implies the existence of an expression hierarchy of the steady state value of a given network node (Villarreal et al., 2012). For example, the case $\alpha_{i}\gg 1$ implies that $\left\langle q_{i}^{st}\right\rangle \to 0$ .

It is worth mentioning that the time evolution of an ensemble may lead to a state of equilibrium, in which the ensemble average do not longer change with time (though individual members of the ensemble keep evolving); this occurs when the ensemble is ergodic, which means that the ensemble average at a fixed time coincides with the time average taken over the dynamical evolution of an individual of it: $\left\langle q_{i}\left(t\right)\right\rangle ={\bar{q_{i}\left(t\right)}}^{t}$ , where ⟨⋅⟩ denotes the ensemble average, and the overline the time average.

### 2.3 Numerical methods

A *Python* interactive program was coded to integrate the stochastic differential equation system, also implementing an interactive interface to directly modify the initial conditions using the packages numpy, scipy, matplotlib and ipywidgets. For the computation, the differential equations system were solved using a collection of numerical algorithms for integrating Ito and Stratonovich stochastic ordinary differential equations (SODEs). In this study we used the Euler-Maruyama algorithm for Ito equations. The list of functions, logic rules, stochastic differential equations and diagram files used on this work can be consulted in the GitHub repository https://github.com/DrDavidMM/Stochastic with further instructions for use. For the results, all conditions were iterated 10,000 times. However, for illustrative purposes most plots in the figures were composed considering 10 iterations. The *Python* source code for the mathematical model presented in this work is available upon request.

## 3 Results

### 3.1 Effect of stochastic perturbations on the CD4 T cell differentiation process

**FIGURE 4:**
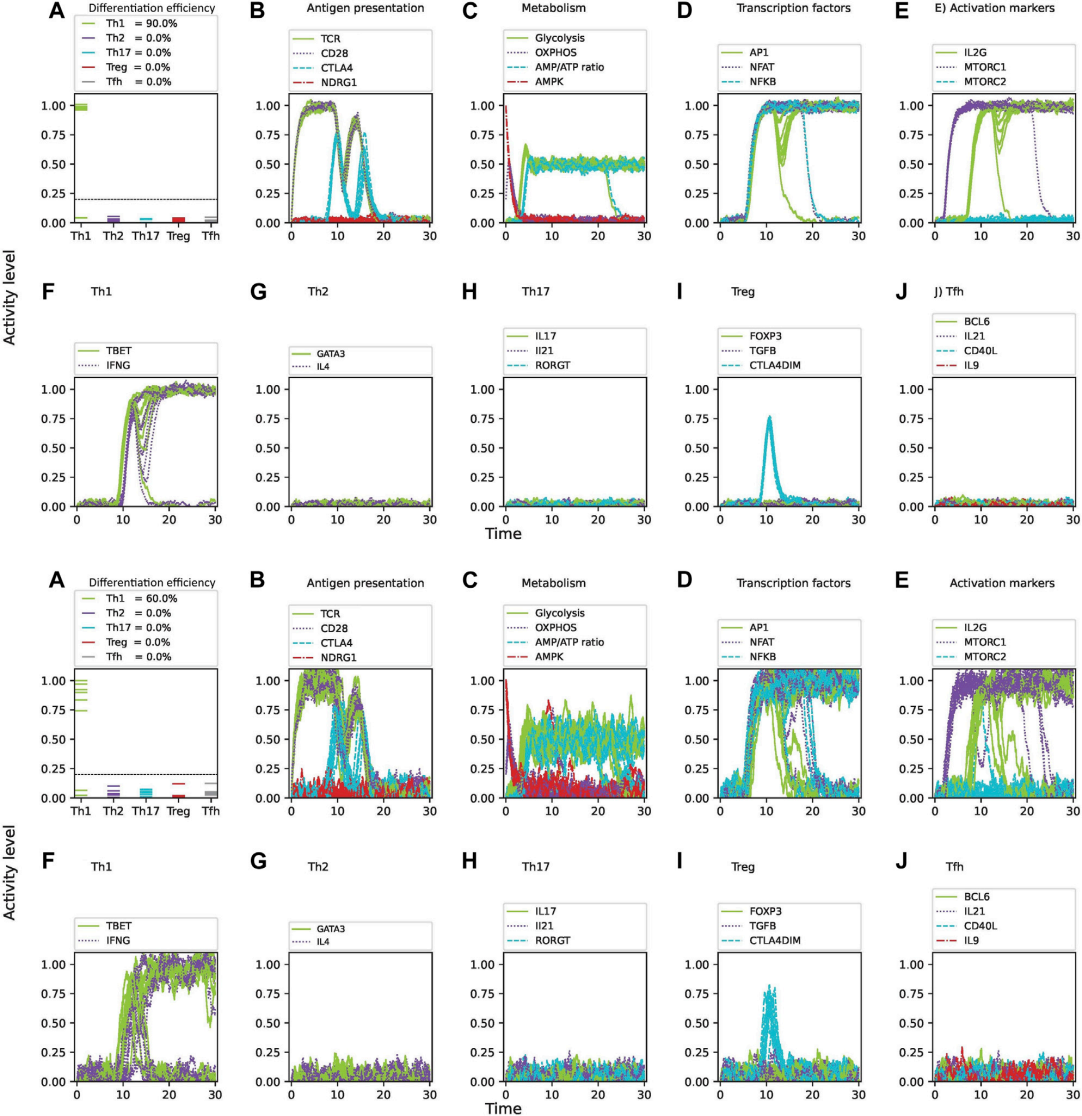
Intrinsic noise significantly affects the differentiation efficiency of CD4 T cells under optimal levels of Th1-inducing cytokines (IFN- γ , IL-12, IL-18, IL-33), nutrients (glutamine, tryptophan), and oxygen availability. Upper panel: 3 % noise. Lower panel: 10 % noise. **(A)** Fraction of differentiated cells expressed as the level of activity of lineage-defining transcription factors at 30 units of time after T cell activation (TCR stimulation and CD28 costimulation). The dotted horizontal line represents a threshold of activation level where activation levels below the value 0.2 are considered as not differentiated. **(B–E)** Activity levels of key molecules and pathways involved in CD4 T cell activation and differentiation: **(B)** antigen presentation and regulatory elements, **(C)** metabolism, **(D)** transcription factors, **(E)** activation markers. **(F–J)** Activity of T cell lineage-defining transcription factors and production of specific cytokines over time.

The effect of random perturbations of either the cell micro-environment or the intracellular interactions themselves on the network dynamics was analyzed through a set of coupled stochastic differential equations describing the rate of change of the expression level of the network constituents. Different levels of noise were introduced into the equations and 10,000 iterations were performed to obtain an average percentage of differentiated cells under each assayed condition. Differentiation efficiency here refers to the fraction of cells reaching optimal expression levels of phenotype-specific transcription factors and their corresponding cytokines. This parameter is denoted here by ε , and may vary in the range 0≤ε≤1 . The case with ε=1 corresponds an idealized situation in absence of noise. Figure 4 shows the dynamics of CD4 T cell activation and differentiation under Th1 cytokine conditions and optimal nutrient levels considering 3 % and 10 % noise. Results shows that differentiation efficiency is sensitive to noise. At a baseline noise level of 3 % , we observed that ε=0.85 for Th1 cell differentiation. Increasing noise levels to 10 % reduced the differentiation efficiency to approximately ε=0.60 for Th1 cells. The analysis was then applied to obtain differentiation efficiencies under Th1, Th2, Th17, Treg and ${\text{T}}_{FH}$ cytokine conditions. Table 1 summarizes the average differentiation efficiencies under 3 % , 5 % and 10 % noise levels. The noise level reduced the differentiation efficiency similarly for all phenotypes. Small fractions of phenotypes different from Th1 were observed approximately at 15 % noise. Plotting the iterations allows to observe the fluctuations in the activity of nodes such as antigen presentation, metabolism, transcription factors, activation markers and cytokine production (Figures 4B–J). It can be observed that the activity of Th1 defining elements such as T-bet and IFN- γ production reaches stable optimal values for most iterations, whereas a relatively small fraction decays after a transient expression. Accordingly, key factors such as metabolism and transcription factors exhibit a corresponding decay. Notably, the fraction of decaying factors increases with higher noise intensities.

**TABLE 1:** Effect of three noise levels on CD4 T cell differentiation. Averages obtained from 10,000 iterations for each case are shown.

Cytokine condition	3% Noise	5% Noise	10% Noise
Th1	0.87	0.76	0.62
Th2	0.85	0.74	0.60
Th17	0.85	0.76	0.61
Treg	0.86	0.78	0.60
${\text{T}}_{FH}$	0.85	0.76	0.62

**FIGURE 5:**
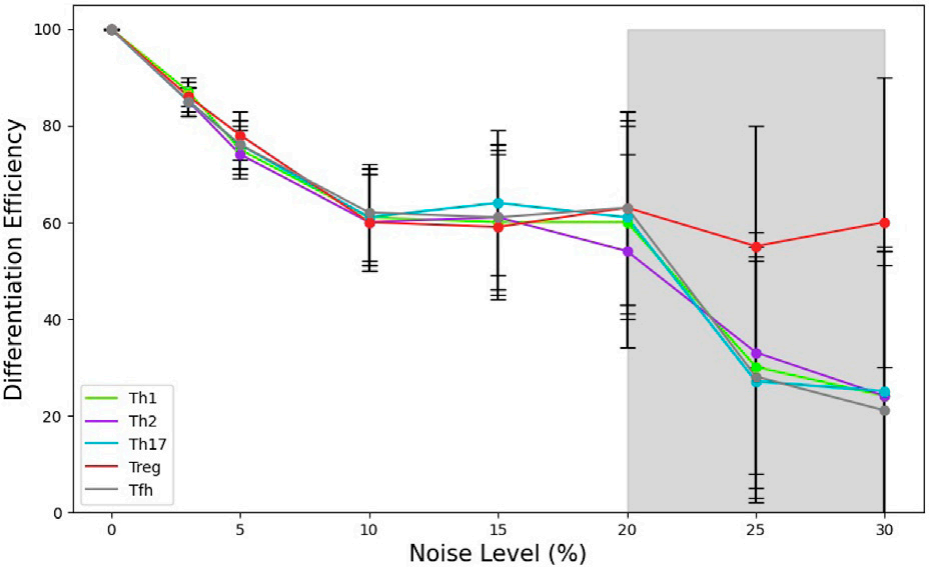
Relationship between the noise level and CD4 T cell differentiation efficiency, ε , under initial Th1, Th2, Th17, Treg and ${\text{T}}_{FH}$ cytokine conditions. Each point represents the average of 10,000 iterations. From an initial value ε=1.0 in absence of noise, this parameter decreases linearly in the small noise interval, 0−10% , developing a plateau around ε∼0.6 , for noise levels in the range of 10–20 % . For higher noise levels, we observe a subsequent decrement of the average efficiency for all T-helper cell subtypes, except for Treg which shows a more stable behaviour, keeping a value ε∼0.6 .

In Figure 5 we present the changes induced by different noise levels on the T cell differentiation efficiency under a specific cytokine microenvironment: Th1, Th2, Th17, Tref or ${\text{T}}_{FH}$ . For relatively small noise levels, ≤10% , we observe that ε decreases linearly with increasing noise, down to a value ε∼0.6 . Notably, enhancing noise levels between 10 % and 20 % , do not induce a further decrement of ε , but this parameter shows a plateau at such value. Afterwards, increasing noise levels with values >20% , causes a drastic reduction of the differentiation efficiency for all considered phenotypes, except for Treg. Thus, a level of noise around 20 % can be interpreted as a threshold beyond which most of the specific phenotype expression is significantly reduced.

**FIGURE 6:**
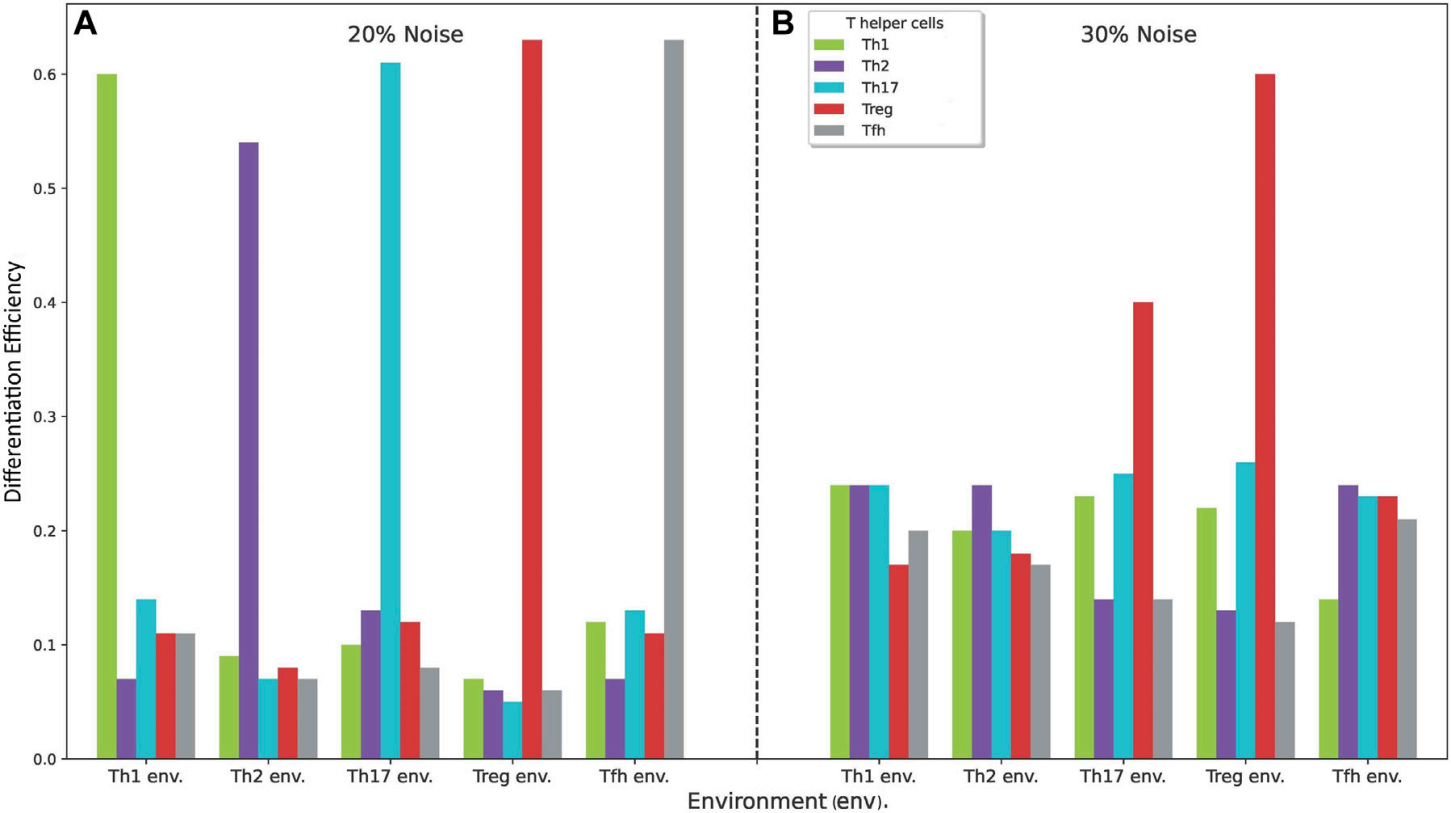
Phenotypic profiles generated under phenotype-specific cytokine environments (Th1, Th2, Th17, Treg and ${\text{T}}_{FH}$ ) under **(A)** 20 % and **(B)** 30 % of noise. In the first case, the corresponding phenotype prevails with differentiation efficiencies ε∼0.5−0.6 , whereas other phenotypes are less expressed (ε∼0.05−0.1) . In the second case, there is no prevailing phenotype at Th1, Th2, Th17 and ${\text{T}}_{FH}$ cytokine conditions (with ε∼0.1−0.25 ). However, a moderate predominance of Treg (ε∼0.4) is obtained in the Th17-cytokine microenvironment, while a marked predominance of Treg (ε∼0.6) is obtained in its phenotype-specific environment in spite of the high noise level.

Figure 6 shows the phenotypic profiles generated under phenotype-specific cytokine environments (Th1, Th2, Th17, Treg and ${\text{T}}_{FH}$ ) under the presence of 20 % and 30 % of noise. Figure 6A shows that the corresponding phenotype still predominates at 20 % of noise, with a differentiation efficiency ε∼0.6 , while populations pertaining to other phenotypes are expressed with low values ε∼0.1 . Instead, at 30 % of noise, populations pertaining to different effector phenotypes become similarly populated, with ε∼0.1−0.2 for Th1, Th2, and ${\text{T}}_{FH}$ microenvironments so that the predominance of the specific phenotype is lost. In contrast, in the case of the Th17 and Treg environments the Treg phenotype predominate (with respective efficiencies ε∼0.4 and ε∼0.6 ) even at high noise levels (Figure 6B).

### 3.2 Effect of stochastic perturbations under variable nutrient and oxygen conditions

Immunological challenges such as viral infections or autoimmune diseases induce a variety of cytokines that does not necessarily match definite patterns inducing particular effector phenotypes. Rather, the presence of infectious agents, and particularly during their persistence in the host, induces the production of mixtures of cytokines that, in principle, may act in a synergic or antagonistic way in the stimulation of naïve cells (reviewed in (Dupage and Bluestone, 2016). The multiple influences converging on phenotype-defining transcription factors are illustrated in Figure 2. Moreover, availability of nutrients and oxygen has a direct effect on T cell differentiation in sites of proliferation such as inflammation sites and lymph nodes (Tao et al., 2015; Swamy et al., 2016; Tykocinski et al., 2017). Here we analyze the effect of noise on CD4 T cell differentiation under mixtures of environmental cytokines and changing conditions of glutamine, trypthophan and oxygen availability.

**FIGURE 7:**
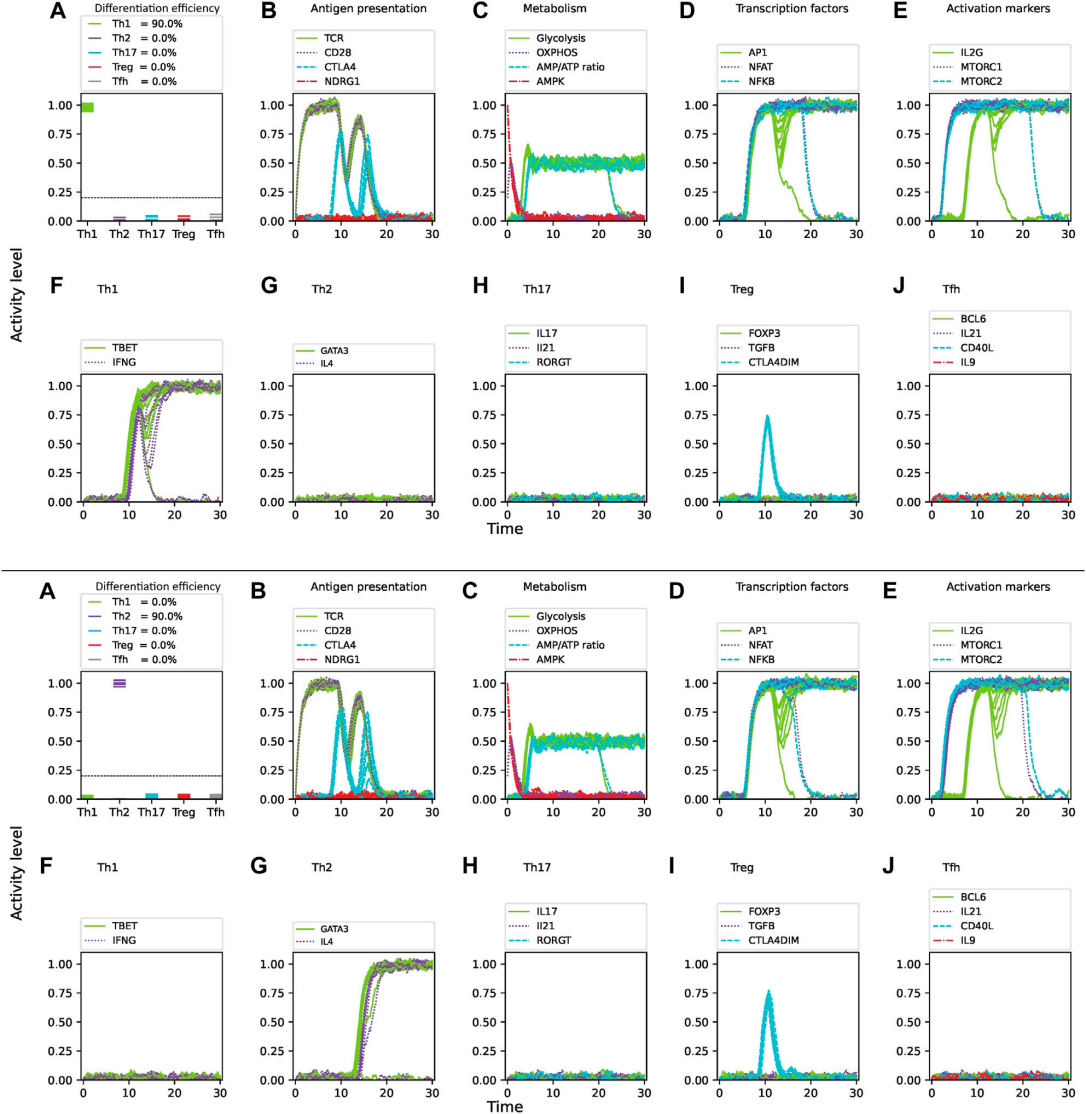
Effect of glutamine deprivation on CD4 T cell activation and differentiation under optimal levels of Th1 (IFN- γ , IL-12, IL-18, IL-33) and Th2 (IL4, IL-33) inducing cytokines, as well as tryptophan and oxygen availability, under 3% noise. Upper panel: Optimal glutamine level. Lower panel: Complete glutamine deprivation. **(A)** Fraction of differentiated cells expressed as the level of activity of lineage-defining transcription factors at 30 time units from activation (TCR stimulation and CD28 costimulation). The dotted horizontal line represents a threshold of activation level where activation levels below the value 0.2 are considered as not differentiated. **(B–E)** Activity levels of key molecules and pathways involved in CD4 T cell activation and differentiation: **(B)** antigen presentation, **(C)** metabolism, **(D)** transcription factors, **(E)** activation markers. **(F–J)** Activity of lineage-defining transcription factors and production of specific cytokines over time.

Glutamine, when metabolized into alpha-ketoglutarate (AKG), enters the mitochondrial citric acid cycle and upregulates mTORC1, enhancing glycolysis (Swamy et al., 2016). Glutamine is required for CD4 T cell activation and induction of the Th1 phenotype, and can promote the expression of the Th1 transcription factor T-bet (Klysz et al., 2015; Swamy et al., 2016). The effect of glutamine deprivation was examined under conditions simulating a mixture of optimal concentration of Th1/Th2 cytokines, as well as optimal nutrient and oxygen levels, and with 3 % noise intensity. Figure 7 (upper panel) shows an average Th1 differentiation ε=1.00 when all nutrients are available. As shown before, using a deterministic model ((Martínez-Méndez et al., 2022)), the removal of glutamine from the initial conditions under a combined Th1/Th2 cytokine environment leads to a bias towards Th2 differentiation Figure 7 (lower panel). This shift underscores the critical role of glutamine in supporting Th1 phenotype development and highlights how nutrient availability can significantly alter T cell fate even in the presence of mixed cytokine signals, as reported (Nakaya et al., 2014). Table 2 shows that increased levels of noise reduce the differentiation efficiency towards the Th1 and Th2 phenotypes under both nutrient conditions (all nutrients and no glutamine). Thus, when a Th1/Th2 cytokine mixture was simulated under optimal nutrient levels, a Th1 differentiation efficiencies of ε=0.84 , 0.71 and 0.59 were obtained using 3 % , 5 % and 10 % noise, respectively, while null or negligible levels of Th2 cells were obtained. Instead, under glutamine deprivation, Th1 differentiation is drastically reduced, and a Th2 differentiation with ε=0.86 , 0.71 and 0.66 was obtained. Thus, modeling is in agreement with experimental results showing that glutamine is able to promote a shift from a Th1 to Th2 effector response (Nakaya et al., 2014).

**TABLE 2:** Effect of three noise levels on CD4 T cell differentiation under different cytokine and nutrient combinations. Averages obtained from 10,000 iterations for each case are shown.

Cytokine condition	3% Noise	5% Noise	10% Noise
Th1 + Th2 + all nutrients	0.84 Th1, 0.00 Th2	0.71 Th1, 0.01 Th2	0.59 Th1, 0.02 Th2
Th1 + Th2 + no glutamine	0.00 Th1, 0.86 Th2	0.0 Th1, 0.71 Th2	0.03 Th1, 0.66 Th2
Th1 + Treg + all nutrients	0.89 Th1, 0.01 Treg	0.69 Th1, 0.07 Treg	0.52 Th1, 0.08 Treg
Th1 + Treg + no tryptophan	0.00 Th1, 0.84 Treg	0.01 Th1, 0.79 Treg	0.02 Th1, 0.68Treg
Th17 + Treg + all nutrients	0.00 Th17, 0.92 Treg	0.02 Th17, 0.75 Treg	0.01 Th17, 0.56 Treg
Th17 + Treg + no oxigen	0.79 Th17, 0.00 Treg	0.72 Th17, 0.01 Treg	0.65 Th17, 0.02 Treg

**FIGURE 8:**
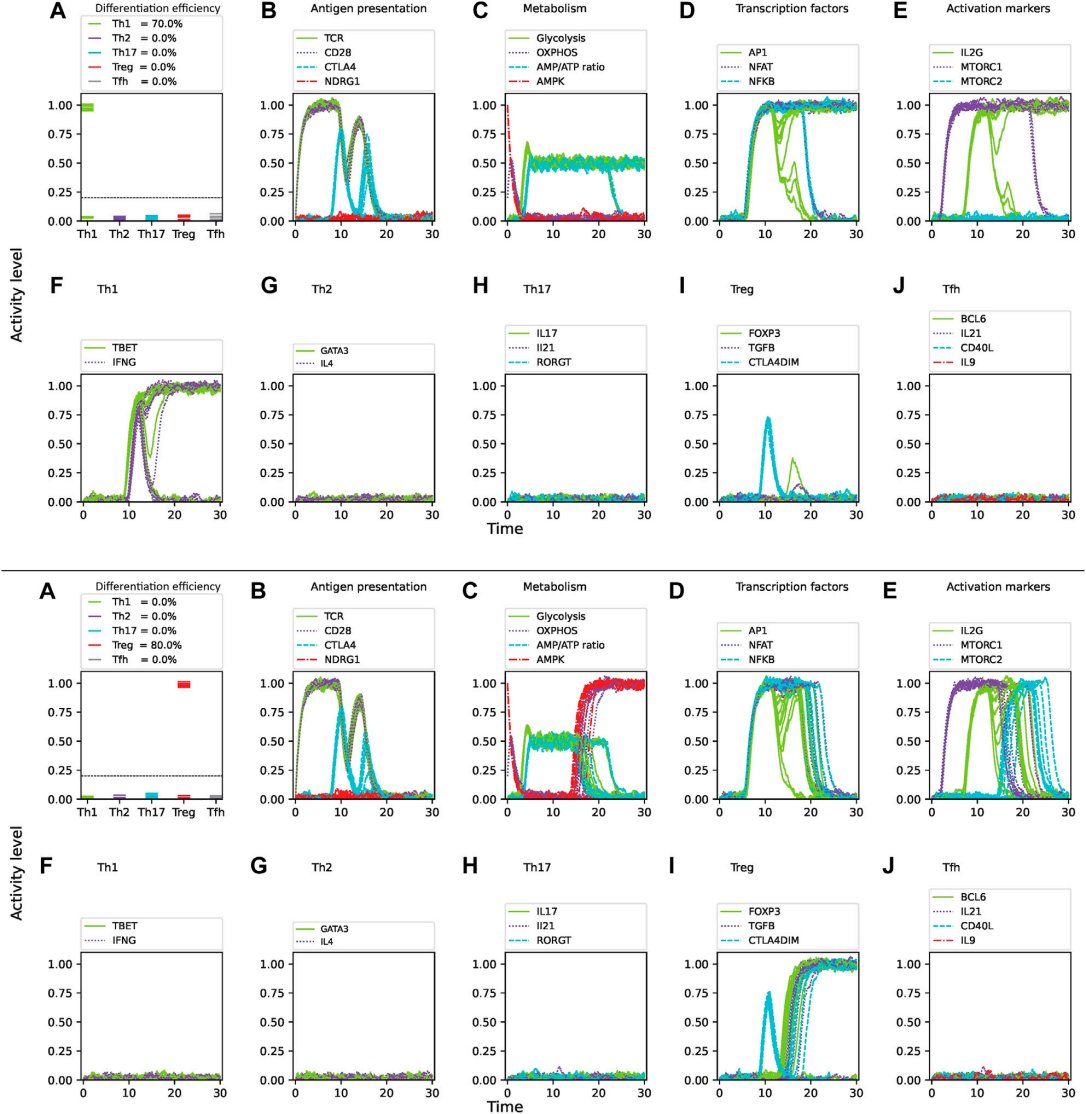
Effect of tryptophan deprivation on CD4 T cell activation and differentiation under optimal levels of Th1 (IFN- γ , IL-12, IL-18, IL-33) and Treg (TGF β , IL-10) inducing cytokines, glutamine, and oxygen availability, subjected to 3% of noise. Upper panel: Optimal tryptophan level. Lower panel: Complete tryptophan deprivation. **(A)** Fraction of differentiated cells expressed as the level of activity of lineage-defining transcription factors at 30 time units from activation (TCR stimulation and CD28 costimulation). The dotted horizontal line represents a threshold of activation level where activation levels below the value 0.2 are considered as not differentiated. **(B–E)** Activity levels of key molecules and pathways involved in CD4 T cell activation and differentiation: **(B)** antigen presentation, **(C)** metabolism, **(D)** transcription factors, **(E)** activation markers. **(F–J)** Activity of T cell lineage-defining transcription factors and production of specific cytokines over time. Notice the metabolic shift from glycolysis to OXPHOS induced by tryptophan depletion.

Tryptophan is an essential amino acid that plays a crucial role in the protein synthesis and proliferation of activated T cells. Notably, studies have shown that tryptophan degradation is used by tumor cells to evade the immune system by depleting its availability for surrounding immune cells, thus hindering their correct differentiation and antitumoral activity (Mellor and Munn, 2004). Tryptophan can activate the ROR γ t and T-bet transcription factors, inducing polarization of CD4 T cells to the Th17 and Th1 phenotypes (Criado et al., 2009; Yan et al., 2010; Tykocinski et al., 2017). On the other hand, the tryptophan catabolism can induce a Treg phenotype (Gargaro et al., 2021). Tryptophan 2,3-dioxygenase (TDO) and Indoleamine 2,3-dioxygenases (IDO1 and IDO2) are tryptophan catabolic enzymes that degrade it into kynurenine. IDO1 is often overexpressed by cancer cells (Mellor and Munn, 2004). However, small-molecule inhibitors such as epacadostat can block IDO1 activity and restore anti-tumoral T cell immunity in mice, in synergy with immune checkpoint inhibitors or cancer vaccines (Peyraud et al., 2022). Simulations using the stochastic model (Figure 8 upper panel) show that in the presence of a mixture of Th1 and Treg cytokines under optimal levels of nutrients, the Th1 phenotype is induced. Deprivation of tryptophan induces a shift from Th1 to Treg (Figure 7 lower panel). Thus, modeling upholds the observation that tryptophan supports differentiation to Th1 even in the presence of Treg-defining cytokines. Modeling also shows that tryptophan depletion shifts the system toward a Treg phenotype. Increased levels of noise reduce the differentiation efficiency towards the Th1 and Treg phenotypes in the presence or absence of tryptophan, respectively (Table 2). Thus, when a Th1/Treg cytokine mixture was simulated under optimal nutrient levels, Th1 differentiation with ε=0.89 , 0.69 and 0.52 was obtained using 3 % , 5 % and 10 % noise, respectively; low levels of Treg cells were then observed ( ε=0.01 , 0.07 and 0.08). Under tryptophan deprivation, Th1 differentiation is drastically reduced and Treg differentiation with ε=0.84 , 0.79 and 0.68 was obtained for 3%, 5% and 10% noise, respectively. These results agree with the observed inhibition of effector immune response under tryptophan deprivation (Gargaro et al., 2021).

**FIGURE 9:**
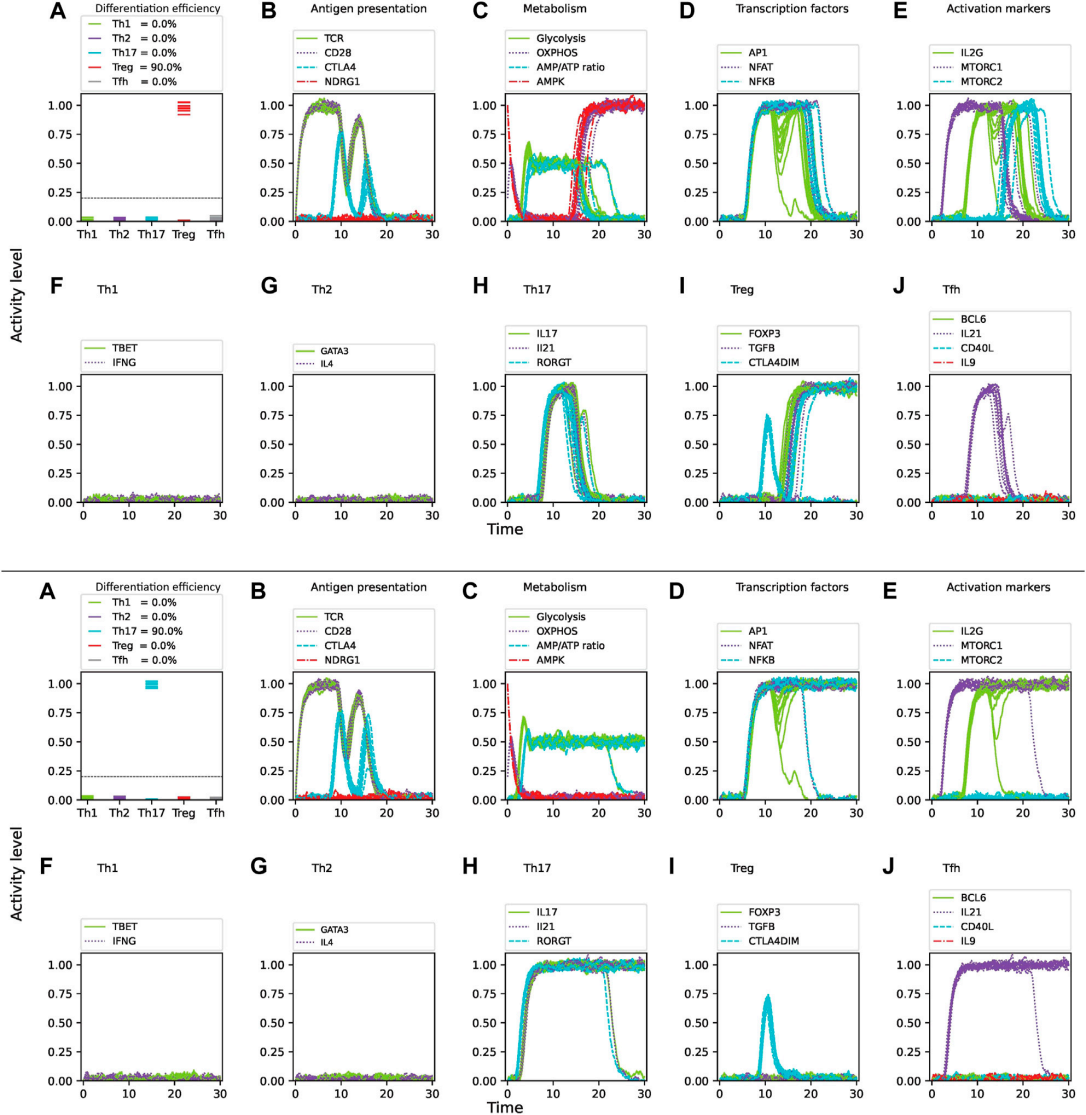
Effect of oxygen deprivation on CD4 T cell activation and differentiation under optimal levels of Th17 (IFN- γ , IL-12, IL-18, IL-33) and Treg (TGF β , IL-10) inducing cytokines and nutrients, subjected to 3% of noise. Upper panel: Optimal oxygen level. Lower panel: Complete oxygen deprivation. **(A)** Fraction of differentiated cells expressed as the level of activity of lineage-defining transcription factors at 30 time units from activation (TCR stimulation and CD28 costimulation). The dotted horizontal line represents a threshold of activation level where activation levels below the value 0.2 are considered as not differentiated. **(B–E)** Activity levels of key molecules and pathways involved in CD4 T cell activation and differentiation: **(B)** antigen presentation, **(C)** metabolism, **(D)** transcription factors, **(E)** activation markers. **(F–J)** Activity of T cell lineage-defining transcription factors and production of specific cytokines over time. Notice the metabolic shift from OXPHOS to glycolysis induced by hypoxia.

Hypoxia is a common characteristic of proinflammatory environments (Dang et al., 2011). The hypoxia-responsive factor 1-alpha (HIF-1 α ) is a vital mediator that responds to hypoxic conditions and influences the T cell metabolism and differentiation. Hypoxia stabilizes HIF-1 α and induces its translocation to the nucleus where it dimerizes and targets the expression of glycolysis, angiogenesis, and apoptosis genes (Semenza, 2000). Activation of HIF-1 α by low oxygen conditions induces the differentiation of Th17 cells and other effector T cell subsets by promoting ROR γ t expression and supporting glycolysis and proliferation (Dang et al., 2011; Oestreich et al., 2015; Miska et al., 2019). The PI3K-AKT-mTOR pathway is crucial for activation of HIF-1 α , especially during persistent antigen stimulation in hypoxic environments (Semenza, 2000). HIF-1 α is downregulated through the ubiquitin-proteasome pathway under normoxic conditions (Wang, 1995). The metabolic switch mediated by HIF-1 α suggests a role for it in the divergence between effector and regulatory subsets. HIF-1 α can negatively regulate Treg differentiation by binding to Foxp3, a key transcription factor that promotes Treg differentiation, directing it for proteasomal degradation (Wang et al., 1995; Dang et al., 2011). Thus, HIF-1 α plays a crucial role in the inhibition of regulatory T cell subsets. These positive and negative interactions influencing the activity of HIF-1 α were included in the stochastic model and the effect of hypoxia was assessed by incorporating 3 % noise. Simulations shows that under a mixed Th17/Treg cytokine condition, optimal levels of oxygen induce Treg differentiation (Figure 8, upper panel). However, hypoxia induces a shift towards a Th17 phenotype under the same cytokine conditions (Figure 9, lower panel). As shown for the previous cases, increasing levels of noise reduces the percentages of Treg and Th17 differentiated cells (Table 2). Thus, when a Th17/Treg cytokine mixture was simulated under optimal nutrient and oxygen levels, Treg differentiation was observed with ε=0.92 , 0.75 and 0.56 by using 3 % , 5 % and 10 % noise, respectively. Under oxygen deprivation, Treg differentiation is drastically reduced and Th17 differentiation with ε=0.79 , 0.72 and 0.65 was obtained. These results are consistent with experimental reports indicating that hypoxia inhibits the Treg phenotype in inflammatory environments, and promotes the predominance of the Th1 and Th17 phenotypes (Dang et al., 2011; Shi et al., 2011; Button et al., 2017).

## 4 Discussion

The function of the immune system embraces inherent variability at the cellular level and microenvironmental conditions. This is evidenced by the profile of CD4 T cell populations generated *in vitro* under controlled polarizing conditions and analyzed flow cytometry, showing that not all cells in a defined population are equally responsive to activation and differentiation stimulus (Espinosa et al., 2020; Siracusa et al., 2021). From a previously characterized continuous deterministic model of CD4 T cell function Martínez-Méndez et al. (2022), here we explored the effect of intrinsic noise on the differentiation efficiency of CD4 T cells into effector phenotypes under defined cytokine and nutrient conditions by translating the deterministic model into a stochastic scheme.

Our results demonstrate that the efficiency of CD4 T cell differentiation is sensitive to intrinsic noise. Increasing noise intensity from 3 % to 10 % resulted in significant reductions in differentiation efficiencies across all examined phenotypes. This result highlights the susceptibility of the underlying network governing T cell fate decisions to stochastic fluctuations. Notably, we observed the emergence of low levels of mixed cell phenotypes, besides the predominant phenotypes, at a threshold defined by 20 % of noise. So, stochastic modeling suggests that the system is resilient to fluctuations up to 20% , where at least a relative fraction of 0.6 of the cells differentiate to the expected phenotype, whereas loss of specific phenotypes is observed at higher noise levels (Figure 6), with exception of Treg. It is possible to suggest that the T-bet, GATA3, ROR γ t, and BCL6 dependence on mTORC1 and mTORC2 for activity, in combination with high levels of noise, has a negative impact on the efficiency of differentiation toward the corresponding phenotypes. Foxp3 expression, instead, is independent of mTOR activation so this may favor a Treg dominance under high stress (noise) conditions (Figure 5). So, resilience of Tregs associates to robustness against noise perturbations up to 30% , in which the differentiation efficiency is ε≈0.6 , implying that most cells differentiate to the expected phenotype.

*In vitro* experiments performed using highly purified naïve CD4 T cells activated through the TCR and CD28 in the presence of specific phenotype-inducing cytokines yield evidence on the effect of intrinsic noise on T cell differentiation. Well-controlled settings assured minimal microenvironmental fluctuations besides the intrinsic biological variability of naïve cells. Analysis of the whole cell population in a single-cell basis by flow cytometry allowed to show that differentiation efficiency is far from the ideal value ε=1.0 for most of phenotypes, since commonly it varied as follows: ε=0.50−0.80 for Th1, ε=0.20−0.40 for Th2, ε=0.60−0.80 for Th17 and ε=0.80−1.00 for Treg (Espinosa et al., 2020). Similar observations were reported in another study (Siracusa et al., 2021). These results are congruent with the presence of intrinsic noise underlying the efficiency of differentiation, so that cells in the population have different capacities to accomplish the full differentiation process. Accordingly, in our model we observed that the efficiency of functional T-cell differentiation lies in a range ε=0.60−0.90 , as shown in Figures 5, 6. Notably, in the aforementioned experiments, Treg cells showed the best *in vitro* differentiation efficiency, which may be related to the highest robustness to noise perturbations of Treg cells pointed out by the model (Figures 5, 6). Both experimental and theoretical observations suggest that Treg cells comply a primordial role in natural selection, that is, a stable regulation of the immune response.

In microenvironments containing cytokine mixtures increasing levels of noise reduces differentiation efficiency and promotes low amounts of alternative phenotypes (Table 2). Thus, modeling shows that activation of CD4 T cells in the presence of glutamine in a mixed Th1/Th2 cytokine environment leads to Th1 differentiation, whereas removal of glutamine skewed the balance towards Th2. This result agrees with experimental observations showing that glutamine deprivation blocked the expression of T-bet under Th1-polarizing conditions but had no effect on the expression of GATA3 under Th2-polarizing conditions. This change in T-bet expression in glutamine-deprived cells was associated with an almost complete absence of IFN- γ secretion by CD4^+^ T cells exposed to Th1-polarizing cytokines. In marked contrast, glutamine deprivation resulted in IL-4 production by cells cultured under Th2-polarizing conditions (Klysz et al., 2015).

Similarly, activation in a medium depleted of tryptophan in a Th1/Treg cytokine environment shifts the differentiation from Th1 mainly towards a Treg phenotype. On the other hand, hypoxia promoted Th17 differentiation while inhibiting Treg in a Th17/Treg cytokine environment. In both cases, noise has a relevant effect on cell differentiation efficiency. Mixed cytokine environments render differentiation efficiencies different from those obtained in phenotype-specific cytokine conditions under the same levels of noise (compare Table 1; Table 2). This could be explained by the combined effect of noise and the multiple positive and negative interactions between nodes (Figure 2). Thus, modeling agrees with a role of glutamine, tryptophan, an oxygen in the maintenance of stable Th1 and Treg phenotypes even under high noise levels (Yan et al., 2010; Shi et al., 2011; Yang et al., 2021). Thus, in conjunction with nutrients, cytokines and oxygen, intrinsic noise has an important role in shaping the immune response.

The accuracy and robustness of computational models are inherently linked to the assumptions and parameters chosen. However, all the relationships between nodes included in the present network are well established facts supported by the literature and have been added step by step in a modular way on the basis of what is currently accepted in the field of the function of the adaptive immune response. The resulting network includes cascade signaling events, redundant rules, positive and negative feedback between nodes, as well as cross-regulation, particularly between nodes representing the lineage-defining transcription factors. All these interactions diversify, reinforce and regulate initial activation and differentiation signals to lead to cell function. Thus, modeling results can be explained by the network topology, which assures that the system will arrive at stable states reflecting cell functionality despite variable levels of biological intrinsic noise. Results are in agreement with experimental observations, indicating that the main assumptions incorporated into the model structure are correct. Further, the model can continue evolving by introducing relevant complementary elements and new discoveries, in order to explore a wider range of hypothetical conditions related to experimental results and clinic observations.

Our approach contributes to validate the utility of computational models in predicting immune cell behavior. Stochastic modeling allows to explore the effect of random perturbations able to alter the topography of the epigenetic landscape leading to functional phenotypic traits.

## Data Availability

Details of the regulatory network considered in this work can be consulted online at https://github.com/DrDavidMM/Stochastic. The interactive model used here can be provided upon request to authors.
