# Supplementary material for: Modeling uncertainty: the impact of noise in T cell differentiation
Source: Front Syst Biol. 2024 Aug 6;4:1412931. doi: 10.3389/fsysb.2024.1412931 (PMC12341952; doi:10.3389/fsysb.2024.1412931)
Supplement: Supplementary file 2 [file DataSheet3.PDF]

Supplemental Material 3. Effect of two noise levels on CD4 T cell differentiation profiles

**Environment**	**Th1 expr. (%)**	**Th2 expr. (%)**	**Th17 expr. (%)**	**Treg expr. (%)**	**Tfh expr. (%)**
20% Noise					
Th1 env.	60.0%	7.0%	14.0%	11.0%	11.0%
Th2 env.	9.0%	54.0%	7.0%	8.0%	7.0%
Th17 env.	10.0%	13.0%	61.0%	12.0%	8.0%
Treg env.	7.0%	6.0%	5.0%	63.0%	6.0%
Tfh env.	12.0%	7.0%	13.0%	11.0%	63.2%
30% Noise					
Th1 env.	24.0%	24.0%	24.0%	17.0%	20.0%
Th2 env.	20.0%	24.0%	20.0%	18.0%	17.0%
Th17 env.	23.0%	14.0%	25.0%	40.0%	14.0%
Treg env.	22.0%	13.0%	26.0%	60.0%	12.0%
Tfh env.	14.0%	24.0%	23.0%	23.0%	21.0%
