# Supplementary material for: Modeling uncertainty: the impact of noise in T cell differentiation
Source: Front Syst Biol. 2024 Aug 6;4:1412931. doi: 10.3389/fsysb.2024.1412931 (PMC12341952; doi:10.3389/fsysb.2024.1412931)
Supplement: Supplementary file 3 [file DataSheet1.PDF]

Supplementary Material 1. Time dependent and fuzzy logic functions for the CD4 T cell differentiation model.

Time dependent functions for antigen presentation and the interaction of CD80/86 peptides with CTLA4 or CD28:

Antigen = AttAnt / (1 + e ** (+ 1 * (t - TAnt)))

CD8086 = AttCD8086 / (1 + e ** (+ 1 * (t - TCD8086)))

Fuzzy logic functions:

WTCR = Antigen * (1 - CTLA4DIM)

WCD28 = CD8086 * (1 - CTLA4DIM)

WAP1 = RASGTPR * (1 - PRED)

WCD25 = IL2G * (1 - CTLA4DIM)

WIL2G = NFAT * AP1 * (1 - NDRG1)

WIL2E = IL2G

WMTOR = (CD25 + AKT) - (CD25 * AKT)

WZAP70 = (TCR * LCK) * (1 - CTLA4DIM) * (1 - PRED)

WSTAT5 = CD25 * (1 - CTLA4DIM)

WNFAT = (CA) * (1 - CS)

WNFKB = PKC * (1 - PRED)

WAKT = (((CD28) * (1 - CTLA4DIM)) + (PDK1)) - (((CD28) * (1 - CTLA4DIM)) * (PDK1))

WCTLA4 = IL2G * ZAP70

WCTLA4DIM = ((CTLA4 * CD8086) + (FOXP3 * TGFB)) - ((CTLA4 * CD8086) * (FOXP3 * TGFB))

WBCL2 = AKT

WNDRG1 = NFAT * (1 - AKT)

WDAG = PLC * PIP2

WSOS = CD28

WRASGTPR = ((LAT * SOS * DAG) + (CD25 * DAG)) - ((LAT * SOS * DAG) * (CD25 * DAG))

WLCK = TCR * (1 - CTLA4DIM)

WPDK1 = (CD25 + CD28 + PIP3) - (CD25 * CD28) - (CD28 * PIP3) - (CD25 * PIP3) + (CD25 * CD28 * PIP3)

WLAT = ZAP70

WPLC = (ZAP70 + CD25) - (ZAP70 * CD25)

WPI3K = (ZAP70 + CD25) - (ZAP70 * CD25)

WPIP2 = (PI3K + PLC) - (PI3K * PLC)

WPIP3 = PIP2

WIP3 = PIP2 * PLC

WCA = IP3

WPKC = DAG

WTBET = (IL33E * IL18E * IL12E * IL2E * IFNGE * MTORC1 * NFKB * NFAT * AP1 * TRP * AKG) * (1 - IL4) * (1 - IL10) * (1 - GATA3)

WIFNG = (TBET * AP1 * NFAT) * (1 - GATA3)

WGATA3 = (IL33E * IL4E * IL2E * MTORC2 * STAT5 * NFAT) * (1 - TBET) * (1 - TGFB) * (1 - IFNG) * (1 - BCL6)

WIL4 = (GATA3) * (1 - TBET) * (1 - IFNG)

WFOXP3 = (((TGFBE * IL10E * NFAT * STAT5 * AP1 * IL2E) + (TGFBE * IL10E * IL10 * CTLA4) + (TGFBE * TGFB)) - ((TGFBE * IL10E * NFAT * STAT5 * AP1 * IL2E) * (TGFBE * IL10E * IL10 * CTLA4)) - ((TGFBE * IL10E * IL10 * CTLA4) * (TGFBE * TGFB)) - ((TGFBE * IL10E * NFAT * STAT5 * AP1 * IL2E) * (TGFBE * TGFB)) + ((TGFBE * IL10E * NFAT * STAT5 * AP1 * IL2E) * (TGFBE * IL10E * IL10 * CTLA4) * (TGFBE * TGFB))) * (1 - IFNG) * (1 - HIF1A) * (1 - IL6E)

WIL10 = TGFBE * FOXP3

WTGFB = FOXP3

WRORGT = (((IL6E * IL21E * TGFBE * AP1 * MTORC1 * TRP) + (IL21E * TGFBE * AP1 * MTORC1 * AKG) + (HIF1A)) - ((IL6E * IL21E * TGFBE * AP1 * MTORC1 * TRP) * (IL21E * TGFBE * AP1 * MTORC1 * AKG)) - ((IL21E * TGFBE * AP1 * MTORC1 * AKG) * (HIF1A)) - ((IL6E * IL21E * TGFBE * AP1 * MTORC1 * TRP) * (HIF1A)) + ((IL6E * IL21E * TGFBE * AP1 * MTORC1 * TRP) * (IL21E * TGFBE * AP1 * MTORC1 * AKG) * (HIF1A))) * (1 - TBET) * (1 - FOXP3) * (1 - GATA3)

WIL21 = (((IL21E * RORGT) + (IL6E * BCL6)) - ((IL21E * RORGT) * (IL6E * BCL6))) * (1 - IFNG) * (1 - IL4) * (1 - IL10)

WIL17 = RORGT

WBCL6 = (IL6E * IL21E * AP1 * MTORC1) * (1 - RORGT) * (1 - TBET) * (1 - GATA3)

WIL9 = BCL6

WCD40L = BCL6

WMTORC1 = (((MTOR * AKT) + (MTOR * AKG)) - ((MTOR * AKT) * (MTOR * AKG))) * (1 - AMPK) * (1 - RAPA)

WMTORC2 = ((MTOR * AMPK) + (MTOR * IL4E)) - ((MTOR * AMPK) * (MTOR * IL4E))

WLKB1 = (AKT * AMPATPratio)

WAMPK = (LKB1 * (1 - MTORC1) + CA * AMPATPratio * (1 - MTORC1) + AKT * AMPATPratio * (1 - MTORC1) + FOXP3 + BCL6 + METF) - ((LKB1 * (1 - MTORC1) * CA * AMPATPratio * (1 - MTORC1)) - (LKB1 * (1 - MTORC1) * AKT * AMPATPratio * (1 - MTORC1)) - (LKB1 * (1 - MTORC1) * FOXP3) - (LKB1 * (1 - MTORC1) * BCL6) - (LKB1 * (1 - MTORC1) * METF) - (CA * AMPATPratio * (1 - MTORC1) * AKT * AMPATPratio * (1 - MTORC1)) - (CA * AMPATPratio * (1 - MTORC1) * FOXP3) - (CA * AMPATPratio * (1 - MTORC1) * BCL6) - (CA * AMPATPratio * (1 - MTORC1) * METF) - (AKT * AMPATPratio * (1 - MTORC1) * FOXP3) - (AKT * AMPATPratio * (1 - MTORC1) * BCL6) - (AKT * AMPATPratio * (1 - MTORC1) * METF) - (FOXP3 * BCL6) - (FOXP3 * METF) - (BCL6 * METF)) + ((LKB1 * (1 - MTORC1) * CA * AMPATPratio * (1 - MTORC1) * AKT * AMPATPratio * (1 - MTORC1)) + (LKB1 * (1 - MTORC1) * CA * AMPATPratio * (1 - MTORC1) * FOXP3) + (LKB1 * (1 - MTORC1) * CA * AMPATPratio * (1 - MTORC1) * BCL6) + (LKB1 * (1 - MTORC1) * CA * AMPATPratio * (1 - MTORC1) * METF) + (CA * AMPATPratio * (1 - MTORC1) * AKT * AMPATPratio * (1 - MTORC1) * FOXP3) + (CA * AMPATPratio * (1 - MTORC1) * AKT * AMPATPratio * (1 - MTORC1) * BCL6) + (CA * AMPATPratio * (1 - MTORC1) * AKT * AMPATPratio * (1 - MTORC1) * METF) + (AKT * AMPATPratio * (1 - MTORC1) * FOXP3 * BCL6) + (AKT * AMPATPratio * (1 - MTORC1) * FOXP3 * METF) + (FOXP3 * BCL6 * METF)) - ((LKB1 * (1 - MTORC1) * CA * AMPATPratio * (1 - MTORC1) * AKT * AMPATPratio * (1 - MTORC1) * FOXP3) - (LKB1 * (1 - MTORC1) * AKT * AMPATPratio * (1 - MTORC1) * FOXP3 * BCL6) - (LKB1 * (1 - MTORC1) * FOXP3 * BCL6 * METF) - (CA * AMPATPratio * (1 - MTORC1) * AKT * AMPATPratio * (1 - MTORC1) * FOXP3 * BCL6) - (CA * AMPATPratio * (1 - MTORC1) * FOXP3 * BCL6 * METF) - (AKT * AMPATPratio * (1 - MTORC1) * FOXP3 * BCL6 * METF)) + ((LKB1 * (1 - MTORC1) * CA * AMPATPratio * (1 - MTORC1) * AKT * AMPATPratio * (1 - MTORC1) * FOXP3 * BCL6) + (LKB1 * (1 - MTORC1) * AKT * AMPATPratio * (1 - MTORC1) * FOXP3 * BCL6 * METF) + (CA * AMPATPratio * (1 - MTORC1) * AKT * AMPATPratio * (1 - MTORC1) * FOXP3 * BCL6 * METF)) - (LKB1 * (1 - MTORC1) * CA * AMPATPratio * (1 - MTORC1) * AKT * AMPATPratio * (1 - MTORC1) * FOXP3 * BCL6 * METF)

WGlycolysis = ((((MTORC1 * GLC) + (HIF1A * GLC)) - ((MTORC1 * GLC) * (HIF1A * GLC))) * (1 - AMPATPratio) * (1 - BCL6))

WGLUTAMINOLISIS = GLN

WAKG = GLUTAMINOLISIS

WOXPHOS = AMPK * FA

WAMPATPratio = Glycolysis * (1 - OXPHOS)

WHIF1A = (1-O2) * AKT
